# Pathological online game use of secondary vocational school students: Current situation and its relation to self-esteem and self-identity

**DOI:** 10.3389/fpsyt.2022.937841

**Published:** 2022-08-15

**Authors:** Lixian Yang, Yuan Chen, Mengxia Zhang, Jinkun Zhang

**Affiliations:** ^1^School of Psychology, Fujian Normal University, Fuzhou, China; ^2^Fujian Ocean Vocational School, Fuzhou, China; ^3^Department of Psychology, School of Education, Fujian Polytechnic Normal University, Fuzhou, China

**Keywords:** secondary vocational school, self-esteem, self-identity, pathological online game use, adolescent

## Abstract

Secondary Vocational School Students are particularly susceptible to online game addiction due to adolescent characteristics and superimposed pressures of academic and employment. Based on the theoretical framework of self-identity and self-esteem, the present research conducted a questionnaire survey using samples of secondary vocational school students to investigate the relationship between pathological online game use (POGU), self-esteem and self-identity. The results showed that 15.56% of secondary vocational students' level of POGU met the diagnostic criteria, and POGU and self-esteem appeared significant differences in gender and family types. Moreover, lower self-esteem and self-identity were associated with higher POGU and self-esteem played a partial mediating role in the relationship between self-identity and POGU. We briefly discussed practical implications of our findings and the future research.

## Introduction

The proportion of online game users accounted for more than half (53.6%) of the overall Internet users as of December 2021 [China Internet Network Information Center (CNNIC), 2022]. What is accompanied by this is the problem of online game addiction that had attracted the attention of many researchers. Although there are currently many different expressions about the concept of online game addiction, such as problematic online game use, excessive online gaming, and pathological online game use (POGU) (1–3), most researchers agree to view online gaming overuse as a potential addiction, which may impair attention and academic performance, cause interpersonal problems, produce a range of emotional problems such as increased loneliness and reduced psychological well-being, and even lead to depression and suicide (4–7). This study adopts Gentile's definition of pathological online game use (POGU), which focuses on one's inability to control his excessive use of online game and the negative impact it has on emotion and school performance, either to escape difficulties or lower their intensity (1).

Because online games provide high enjoyment and interactivity with a delicate reward mechanism (8), they have great appeal to adolescents who lack self-control. In addition, as adolescents, secondary vocational school students are particularly vulnerable to social pressures, lack of social support, and satisfaction of psychological needs (9). Previous cross-sectional studies revealed negative correlation between self-identity and POGU (10–12). Self-identity contains how one evaluates the real self, how one envisions the future self and how one adjusts oneself to adapt to surrounding changes, which tap into the feeling that an individual has of understanding himself or herself (13–15). A person with an established self-identity knows enough about himself and is capable of grasping the meaning of the ideals and values he holds (16).

The concept of self-esteem is centered on self-affirmation and self-identification, which is a type of inner consciousness, and is an important factor in personal growth (17–23). Rosenberg (24) defines self-esteem as an attitude toward oneself, which can be positive or negative. It is when one believes he is good enough when dealing with something or being enraptured in a certain state that one gains self-esteem. A significant negative correlation exists between self-esteem and POGU (25–27), the lower the individual's self-esteem, the more likely they are to be addicted to mobile games. Meanwhile, Lemmens et al. (27) declared that self-esteem could be a significant predictor of later pathological online gaming behaviors with autoregressive structural equation models. Self-esteem is the perceived difference between one's ideal self and one's real self, and it is a protective factor for Internet addiction (28), for high self-esteem can help people maintain more positive attitudes in stressful situations and reduce undesirable behaviors (29), thus people with high self-esteem are expected to be less likely to indulge in online games. Teenagers who attend secondary vocational schools have unique characteristics that set them apart from other teenagers. In most cases, they are diverted from the general education because of their ineligibility for it and are “forced” to enter the vocational education. Currently, vocational education is not well understood by the general public, and there are still some prejudices against secondary vocational school students (30). This social prejudice is a cognitive result of stereotypes, which not only affect the out-group's perception of the in-group, but also how in-group members react to that perception, namely, the meta- stereotype (31, 32). According to social identity theory (33, 34), identity is primarily formed by identifying with the group, and when members of the group compare themselves with out-groups and find themselves to be in disadvantage, their self-assessments are skewed negatively, thus resulting in identity threat that hinders achieving positive self-esteem.

Therefore, we hypothesize that self-identity can predict POGU through self-esteem, i.e., self-esteem plays a mediating role in self-identity and POGU.

## Methods

### Study design and samples

Seven hundred and ninety students were sampled by convenient sampling method from five secondary vocational colleges in Fuzhou, Fujian Province, China. All subjects participated in the experiment voluntarily, and 756 questionnaires were collected, with a recovery rate of 95.70%. After eliminating invalid questionnaires, 720 valid questionnaires were received and the efficiency rate was 91.14%. Among them, 388 (53.89%) were male students and 332 (46.11%) were female students; 139 (19.31%) were only children and 581 (80.69%) were non-only children; 352 (48.89%) were first-year students, 198 were (27.50%) second-year students and 170 (23.61%) were third-year students.

### Materials

Demographic questionnaire. The first part of the questionnaire was to collect demographic data on the subjects, including gender, grade level, family type, and whether they were the only child.

Pathological online game use questionnaire. The second part of the questionnaire is to investigate the addiction to online games. It contained 11 items from Gentile's Pathological Video-Game Use Questionnaire in Chinese version (5) and was demonstrated by previous studies with good reliability and validity conducted with Chinese adolescents (35, 36). This questionnaire is a single-dimensional questionnaire, consisting of a total of 11 question items. Subjects were asked to make a three-point scaled frequency assessment of the situation presented by each item (e.g., do you spend more and more time playing or learning to play online games?), namely 0 = never, 1 = sometimes, 2 = often and were recorded into 0, 0.5, 1 for never, sometimes and often, respectively (37). The total score of these 11 items was summed and the higher the score, the more severe the online game addiction (5). In the current study, the Cronbach's alpha coefficient for this questionnaire was 0.854.

#### Self-esteem scale

The scale was compiled by Rosenberg in 1965. Ji Yifu and Yu Xin translated it into Chinese version (38). There are a total of 10 question items (e.g., “I feel that I have several good qualities”) including 5 positive questions and 5 reverse questions. The four-point scoring method is adopted from 1 (strongly disagree) to 4 (strongly agree), and the total score is 10–40, with items 3, 5, 8, 9, and 10 scored reversely, and the higher the score, the higher the level of self-esteem. However, some studies suggest that item 8 “I wish I could have more respect for myself” carries different cultural implications for Chinese (39), so this study scored it positively. In this study, the Cronbach α coefficient of the questionnaire was 0.751.

#### Self-identity scale

The Self-Identity Scale (SIS) developed by Ochse and Plug in 1986 based on Ericson's theory is widely used in adolescent groups. The scale has a total of 19 problem items (e.g., “I feel my way of life suits me”), and the four-point scoring method is used from 1 (strongly disagree) to 4 (strongly agree). The reverse scoring method is used for the item 1, 2, 4, 8, 9, and 12 to18 with higher scores presenting higher self-identity. Li Yi an and Lou Wenjing tested the reliability and validity of this scale with high school students as subjects, and found that this scale had good reliability and validity. The Cronbach α-coefficient of the overall scale was 0.727 (16). In this study, the Cronbach α coefficient of the questionnaire was 0.774.

### Procedure

This study was conducted class by class. To ensure the accuracy of the survey results, two post-graduate students in psychology served as the experimenters and were trained before the implementation of formal measure. The experimenters explained the instructions to the subjects and asked them to fill in the questionnaire according to their real feelings and experience, and the results would be kept confidential. Afterwards, the subjects were given a professional online questionnaire website link and began to answer the questionnaire, which included four parts, demographic questions, SES, SIS, and POGU questionnaire, respectively. The subjects were thanked and debriefed after completion.

## Results

### Common method bias

In this study, Harman's single-factor test was performed on all items in the questionnaire, and the KMO value was 0.900, and the significance of Bartlett's test was 0.000. Eight factors were extracted from 40 items through principal component analysis (PCA). The variance contribution rate of the first factor is 20.458%, lower than the critical value of 40%, indicating that the common method bias is acceptable.

### Descriptive statistics

Table 1 includes the mean and standard deviation value for all variables. POGU total score measures the tendency of secondary vocational school students to be addicts of online games, where a higher score indicates an even stronger tendency of addiction. According to the delimitation standard of addiction with a total score of ≥5 which means exhibited at least 5 of the 11 criteria (40), a further analysis showed that the number of students whose POGU scores met the criteria was 112, accounting for 15.56% of the total number of students surveyed. As for the self-esteem, its average score is about 27 with SD of 4.606. In terms of self-identity, a total of 66 to 68 points indicates a high level of self-identity, based on an average score between 56 and 58 and a standard deviation between 7 and 8, and total scores less than 49 to 50 suggest a lower self-identity (18). In this study, the average score of self-identity is 51.25, indicating that the overall self-identity of secondary vocational school students is at a relatively low level.

**Table 1:** Descriptive statistics and correlation co-efficient between variables (*M* ± *SD*).

**Variable**	***n***	**PERC**	***M***	***SD***	**1**	**2**	**3**	**4**	**5**	**6**	**7**
1. POGU	–	–	2.499	2.024	1						
2. SE	–	–	26.996	4.606	−0.281**	1					
3. SI	–	–	51.254	7.562	−0.352**	0.645**	1				
4. Grade			–	–	0.041	0.015	−0.007	1			
First	352	48.89									
Second	198	27.5									
Third	170	23.61									
5. Gender			–	–	−0.220**	−0.078*	0.012	−0.004	1		
Male	388	53.89									
Female	332	46.11									
6. FT			–	–	0.068	−0.141**	−0.091*	0.06	0.038	1	
One–parent family	92	12.78									
Two–parent family	603	83.75									
Other (raised by generations)	25	3.47									
7. OCON			–	–	−0.036	−0.021	−0.042	−0.018	0.143**	−0.112**	1
Yes	139	19.31									
No	581	80.69									

POGU, pathological online game use; SE, self-esteem; SI, self-identity; FT, family type; OCON, only child or not.

**p < 0.01, *p < 0.05.

### Analysis of differences in demographic variables

There were significant differences in POGU behavior between different genders, with boys significantly higher than girls (*p* < 0.01, Cohen's *d* = 0.451); the same tendency could be found in self-esteem (*p* < 0.05, Cohen's *d* = 0.157), with boys' average score significantly higher than that of girls. But gender didn't differ significantly on self-identity scores. Also, significant difference could be drawn among family types on POGU and self-esteem but not on self-identity to the extent that average scores of students from other families were significantly higher than those from single-parent and two-parent families on POGU (*p* < 0.05, η^2^_*p*_ = 0.010) and students from two-parent families scored significantly higher than single-parent families and other types of families on self-esteem (*p* < 0.01, η^2^_*p*_ = 0.021). While “only child or not” and grade level didn't differ significantly on each of the three independent factors.

### Correlation analysis

Through correlation analysis, the Pearson correlation coefficient of variables including POGU, self-esteem, self-identity, grade, gender, family type, and only child or not of secondary vocational students is obtained, as shown in Table 1.

The findings were consistent with previous studies conducted with other groups, namely, POGU was negatively associated with self-esteem (*r* = −0.281, *p* < 0.01); the statistically significant positive correlation was also found between self-identity and self-esteem (*r* = 0.645, *p* < 0.01). POGU was negatively correlated with self-identity (*r* = −0.352, *p* < 0.01).

### Regression analysis

A stepwise regression analysis was conducted in SPSS24.0 to analyze the data, and the results are shown in Table 2. Gender, grade, family type and “only child or not” were incorporated into the models as control variables in three models. The results in model 1, in which self-identity as the independent variable and POGU as the dependent variable, showed that self-identity had a significant negative relationship with POGU (β = −0.346, *p* < 0.001). In model 2 where self-identity as independent variable and self-esteem as the independent variable, the results suggested that higher self-identity correlated with higher self-esteem (β = 0.639, *p* < 0.001). In model 3, self-identity and self-esteem was jointly added into the independent variables and POGU served as dependent variable. It revealed that there was a significant negative relationship between self-esteem and POGU (β = −0.122, *p* < 0.01). Therefore, the results demonstrated that, for secondary vocational school students, self-esteem serves as a mediator between self-identity and POGU. At the same time, self-identity still has a negative relationship with POGU (β = −0.268, *t* = 3.74, *p* < 0.001), indicating that self-identity not only directly affects POGU, but also affects POGU behavior indirectly through self-esteem. In other words, lower the level of self-esteem and self-identity, the stronger the POGU behavior.

**Table 2:** Linear regression analysis on the relationship between self-esteem and self-identity of secondary vocational students on video game addictive behavior (*n* = 720).

**IV**	**Model 1 (DV: POGU)**		**Model 2 (DV: self–esteem)**		**Model 3 (DV: POGU)**	
	**β**	***t***	**β**	***t***	**β**	***t***
Gender	−0.215	−6.258***	−0.084	−2.939**	−0.226	−6.546***
Grade	0.035	1.025	0.024	0.850	0.038	1.116
FT	0.041	1.185	−0.080	−2.792**	0.031	0.901
OCRN	−0.015	−0.419	0.009	0.312	−0.013	−0.389
SI	−0.346	10.123***	0.639	22.480***	−0.268	−6.028***
SE					−0.122	−2.723**
VIF	1.000		1.000		1.711	
D–W	2.081		2.123		2.097	
R^2^	0.174		0.430		0.183	
F	30.101		107.668		26.545	

IV, independent variable; DV, dependent variable; POGU, pathological online game use; FT, family type; OCON, only child or not; SI, self-identity; SE, self-esteem.

**p < 0.01,***p < 0.001.

### Mediating effect analysis

**Figure 1:**
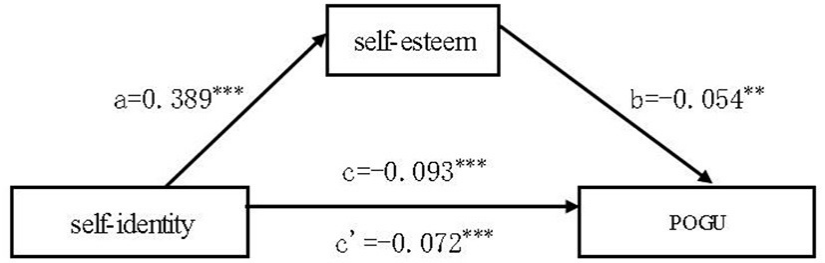
Model of the mediating role of self-esteem between self-identity and POGU (pathological online game use), ***p < 0.001, **p < 0.01. c: direct effect of self-identity on POGU; c': total effect of self-identity on POGU.

The mediation effect test was conducted in PROCESS Procedure for SPSS 3.5 with number of bootstrap samples set for 5,000 (41). In this study, self-identity was used as the independent variable, self-esteem as the mediating variable, and POGU as the dependent variable with gender, grade, family type and “only child or not” as covariates, and the results of the data were obtained in Tables 3A,B. It can be seen from Table 3A that self-identity still has a direct negative relation with POGU, but the effect is weakened. The regression coefficient is −0.072, the mediating effect coefficient is −0.021, and the lower and upper limits of the 95% confidence interval for the mediating effect of self-esteem (−0.039, −0.003) does not contain 0, indicating that self-esteem has a partial mediating effect between self-identification and video game addictive behavior. According to Table 3B and the formula of the effect share: ab/c, it can be calculated that this mediating effect accounts for 22.581% of the total effect. Finally, the model formula can be derived as: POGU = 8.729–0.054^*^ self-esteem-0.072^*^ self-identity. The model was plotted according to the results (see Figure 1).

**Table 3A:** Test results of the mediating effect of self-esteem.

**Effect model**	**Coeff**	***se***	**BootLLCI**	**BootULCI**	**Relative coeff**
Total effect	−0.093	0.009	−0.111	−0.075	
Direct effect (SI = > POGU)	−0.072	0.012	−0.095	−0.048	77.419%
Indirect effect (SI = > SE = > POGU)	−0.021	0.001	−0.039	−0.003	22.581%

POGU, pathological online game use; SI, self-identity; SE, self-esteem.

**Table 3B:** Test results of the mediating effect of self-esteem.

**Dependent variable**	**Independent variable**	***R^2^***	***F***	**coeff**	***se***	***t***
POGU						
	Self-identity	0.174	30.101	−0.093	0.009	−10.123***
Self-esteem						
	Self-identity	0.430	107.668	0.389	0.017	22.48***
POGU						
	Constant			8.729	0.639	13.655***
	Self-identity	0.174	30.101	−0.072	0.012	−6.028***
	Self-esteem	0.183	26.545	−0.054	0.020	−2.723**

POGU, pathological online game use.

***p < 0.001, **p < 0.01.

## Discussion

The survey found that 15.56% of the sample of secondary vocational school students met the diagnostic criteria of online game addiction. Compared with high school students, secondary vocational students face both academic and employment pressure in the process of developing self-awareness, which may make them more eager to find ways to relieve their stress. On the other hand, as has been well documented, the vivid virtual world created by video games can make people temporarily stay away from real-world anxiety and gain the joy of gaming (8, 42, 43). Due to the lack of self-control, secondary school students are especially prone to getting caught up in gaming and unable to extricate themselves. Through the analysis of the POGU behavior within different demographic variables, we also found there are no significant differences in grade level, child status, and family income, while significant differences in genders and family types. The average score of POGU was higher in the male students than in the female, which is consistent with the previous research (44, 45). In a large-scale survey on the online game addiction behavior of teenagers from primary school students to college students, researchers found the proportion of girls addicted to online games was lower than that of boys (44). Ustinaviciene et al. (45) also discovered the same tendency and illustrated that POGU was related to game type and the time spent on it. Namely, male students who played action or combat games were more likely to become addicted than those who played logic computer games, whereas female students who played games for more than 5 h a month were more inclined to become addicted. Several studies have replicated this findings, for example, a recent study of game use among 5,607 Norwegian adolescents showed a significant gender difference in game use, with boys being five times more likely than girls to start playing since age 14 and girls are using social media more often (46). Game type preference also shows gender differences and can be predicted by psychological characteristics, for example, girls' positive feelings about themselves make them more likely to choose First-Person Shooters as their favorite game genre, while boys' internalization difficulties may predict a lower choice of Massively Multiplayer Online Role Playing Games (MMORPGs) (47). Meanwhile, male's playtime was fully mediated by their higher role-playing and shooter games preferences (48). In terms of family type, the average score of online game addiction among students who are in skip-generation raising is significantly higher than that of two-parent and single-parent families. Parental involvement in the use of online games is crucial, and children's use of these games requires greater attention, support, and supervision of their part, and skip-generation raising shifts more responsibility for children from parents to grandparents, leading to the lack of parental education which closely related to adolescent game addiction,for example, in a review study, researchers found that parent-child relationships were strongly associated with problematic game addiction in adolescents, and paternal relationships could serve as a protective factor for children's game addiction (49).

In the demographic analysis of self-esteem, it is found that there is no significant variability across grades and whether they were only children, but there is significant variability across gender, family type and monthly household income. The finding on gender and grade is different from that of Zhang Yan et al. (50) on the analysis of variance. Their study indicated that in secondary vocational technical schools, girls reported greater self-esteem than boys and first-year students scored higher than their older counterparts. Based on the results of the current survey, it has been found that the level of self-esteem of male and female students is significantly different with male students tending to have a higher average level of self-esteem than female students. The imbalance in self-esteem for different genders may be, in part, due to the traditional view suggested by Chen Xuehong et al. (51) in their study that men, presumed to be the mainstay of the society, supposed to possess more good qualities and pay more attention to the internal verification of themselves than females. The same trend was also confirmed in other groups of adolescents,for example, Moksnes and Espnes (52) conducted a survey of 1,239 Norwegian teenage students and found that boys had higher levels of self-esteem and life satisfaction than girls. Aremu et al. (53) indicate that although there is no significant difference in self-esteem levels between boys and girls, more girls reported low self-esteem scores. Regarding the difference in family type, the level of self-esteem of secondary vocational school students in two-parent families was significantly higher than that of the other family types. It shows that parental companionship plays an important role in children's personality improvement. Self-esteem is structured by the intimate relationship with parents (54), and previous studies have also shown that children in divorced families have lower levels of self-esteem than those in intact families (55), and even children in joint physical custody report higher self-esteem than those living exclusively with one parent (56). In terms of monthly family income, compared to students from low-income families, students from high-income families have higher self-esteem levels. This is consistent with research on general high school students' self-esteem: students from high-income families may have higher self-esteem levels than students from low-income families (57).

A relatively low level of self-identity was found among secondary vocational school students. The society has certain prejudice against secondary vocational school students and assumes that only those who are not capable enough, namely got unsatisfactory grades in their high school-entrance examination, will come to these schools. This perspective further leads to the students' disapproval of their own ability and identity as a member of secondary vocational school students, leaving them a negative attitude toward their own, thereby causing low self-identity. Secondary vocational school students yearn for seeking self-identity in online games and they are eager to release their real selves in the game in which they project their emotions onto specific roles, the avatar, without sticking to the restrictions of the real world but to freely express themselves, either to get emotional satisfaction or to escape from the disorder in reality. In this way, they realize their self-worth through the construction of the role identity, their perceived self-identity (58), and if the players get satisfaction from the game, they are more likely to continue game usage (10). Meanwhile, there are also neural mechanism studies that reveal that the phenomenon of higher identification with game avatars in online game addicts may be related to self-concept impairment (59), for example, Leménager et al. (60) found higher activation levels in the left angular gyrus, a brain region that has also been shown to be strongly associated with self-identity,when addicted players perceived game avatars compared to perceived themselves. The same evidence has been confirmed by other researchers (61). The analysis of the differences in self-identity in terms of demographic variables also found that while no significant differences were observed among gender, grade, family type, or whether they were only children, the monthly household income per capita showed significant variation. This suggests that family socioeconomic status and the level of self-identity are related potentially.

Meanwhile, it shows that self-identity is also positively associated with self-esteem. Students with higher self-identity understand themselves better and act in school and life in a more optimistic manner. Consequently, they are able to appreciate themselves more, evaluate themselves more positively and have higher levels of self-esteem. In contrast, a negative outlook is prevalent among students with low self-identity, and they often feel useless and uncertain of their roles, thus developing low self-esteem. Further, self-identity and self-esteem both correlate negatively with POGU. From Erikson's theory of stages of psychological development (62) and Maslow's need - hierarchy theory (63), self-esteem and self-identity play a key role in building a solid personality. High self-esteem and self-identity enable people to make a comprehensive assessment and analysis of themselves and people with high self-esteem and self-identity are better at handling situations that may come their way and finding enjoyment and meaning in life, thus be less likely to become addicted to online games. However, it may be demanding for those who lack self-esteem to handle pressure and problems in life and they are compelled to flee from reality and seek a sense of belonging and accomplishment from the virtual world of online games. This inappropriate “sense of belonging and accomplishment” will make secondary vocational school students deeply immersed in online games and unable to extricate themselves.

## Conclusion

The correlation analysis results of POGU, self-esteem and self-identity of secondary vocational school students show that there is a significant negative correlation between POGU and self-esteem and self-identity, and a significant positive correlation between self-esteem and self-identity. Regression analysis showed self-esteem played a mediating role between self-identity and POGU.

Based on regression analysis of the effects of self-esteem and self-identity on POGU behaviors, we also found that although both self-esteem and self-identity significantly and negatively predicted online game addictive behaviors, the coefficient of *R*^2^ was small, indicating that the explanatory rate of them on online game addictive behaviors was low. This result may be caused by many factors which contribute to addiction to online games in proportion. Previous studies have found many influencing factors of addiction to online games, such as parental support (25, 64, 65), school belonging (66), boredom proneness (67) and so on. In view of this, other relevant variables should be considered for further research in future studies.

The mediating effect of self-esteem is analyzed, and it is found that self-esteem plays a partial mediating role between self-identity and addiction to online games, which means that self-identity may also affect the addictive behavior of online games through other factors. Further research and discussion can be made in the future. From data obtained from the study, it suggests the obvious influence of self-esteem and self-identity on pathological online game use online game, and also provides direction and ideas for formulating relevant educational strategies.

## Data availability statement

The raw data supporting the conclusions of this article will be made available by the authors, without undue reservation.

## Ethics statement

The studies involving human participants were reviewed and approved by Institutional Review Board of the School of Psychology, Fujian Normal University. Written informed consent to participate in this study was provided by the participants' legal guardian/next of kin.

## Author contributions

LY was responsible for the conceptualization. YC finished the data curation and methodology design. MZ finished the original draft under supervision of JZ. All authors contributed to the article and approved the submitted version.

## Funding

This work was supported by the Key Research Institute of Humanities and Social Sciences ofMinistry of Education, China (Grant No: 16JJD190004) and also partly supported by the Fujian Social Science Planning Project under Grant (FJ2018C093).

## Conflict of interest

The authors declare that the research was conducted in the absence of any commercial or financial relationships that could be construed as a potential conflict of interest.

## Publisher's note

All claims expressed in this article are solely those of the authors and do not necessarily represent those of their affiliated organizations, or those of the publisher, the editors and the reviewers. Any product that may be evaluated in this article, or claim that may be made by its manufacturer, is not guaranteed or endorsed by the publisher.
